# Deubiquitylating Enzymes in Hepatocellular Carcinoma

**DOI:** 10.7150/ijbs.113193

**Published:** 2025-06-20

**Authors:** Xingyu Mi, Qingfeng Li, Guo Long, Yilin Pan, Yulin Xie, Shiqi Lu, Liang Xiao, Jianing Tang, Ledu Zhou

**Affiliations:** 1 Department of Liver Surgery, Xiangya Hospital, Central South University, 110 Xiangya Road, Changsha, Hunan 410078, China.; 2 Department of General Surgery, The Second Hospital of Liling City, Baitutang Town, Liling City, Zhuzhou City, Hunan 412207, China.

**Keywords:** hepatocellular carcinoma, deubiquitylating enzymes

## Abstract

Ubiquitination is a reversible and dynamic process, precisely regulated by ubiquitin-activating enzymes (E1), ubiquitin-conjugating enzymes (E2), ubiquitin ligases (E3), and deubiquitinating enzymes (DUBs). Dysregulation of DUBs disrupts the dynamic balance of ubiquitination, contributing to the development of various diseases, particularly cancer. An increasing number of studies have identified dysregulation of DUBs in various tumor types and have explored their regulatory mechanisms in these contexts. Hepatocellular carcinoma (HCC), the most common form of liver cancer, is highly malignant and has limited treatment options, necessitating the exploration of additional therapeutic strategies. Current research has identified dysregulation of DUBs in HCC, but their mechanisms of action remain poorly understood. Given the large number of DUB family members, there is significant potential for further investigation. This review summarizes the DUBs associated with HCC, including their structures, functions, and the mechanisms through which they regulate HCC development. Furthermore, we provide a brief overview of the discussed DUBs, aiming to offer new perspectives for future HCC research.

## Introduction

Ubiquitination is an important post-translational modification process in which ubiquitin molecules, consisting of 76 amino acids, are covalently conjugated to target proteins^1^. The main steps of ubiquitination are mediated by three types of enzymes: ubiquitin-activating enzymes (E1), ubiquitin-conjugating enzymes (E2), and ubiquitin ligases (E3)^2^, ^3^. And deubiquitinating enzymes (DUBs) can remove ubiquitin from substrate proteins. It is noteworthy that ubiquitination is a reversible and dynamic process, precisely and orderly maintained by E1, E2, E3 and DUBs. Ubiquitin proteins are first activated by E1, a process that requires ATP. Subsequently, Ubiquitin proteins bind to E2 and then to substrate proteins through E3 to form a covalent bond^4^. While DUBs can clean ubiquitin proteins from substrate proteins to negatively regulate this process^5^. There are some current researches indicating that imbalance between ubiquitination and deubiquitination is associated with the occurrence and development of human cancer^6^-^10^. Some targeted drugs for the ubiquitin system have been developed and have shown progress^11^. However, considering the number and diversity of E2s, E3s, and DUBs, there is still potential for the development of more drugs. In particular, DUBs are inherently attractive as potential drug targets^11^, ^12^.

Liver cancer is one of the most common malignant tumors of the digestive system. According to the global cancer statistics of 2022 released by the International Agency for Research on Cancer, the mortality rate of liver cancer ranks 3rd^13^. The most common subtype of liver cancer is hepatocellular carcinoma (HCC), accounting for approximately 90% of cases^14^. Curative surgery is the only treatment that has the opportunity to cure early-stage HCC, but the majority of patients are diagnosed at an advanced stage and lose the opportunity for surgery^15^. Although targeted drugs for the treatment of advanced HCC, such as sorafenib and lenvatinib, which are tyrosine kinase inhibitors, have been developed and utilized in clinical practice, there are currently no drugs targeting DUBs used in the treatment of advanced HCC.

This article will review the research progress of deubiquitinating enzymes in HCC, explore their potential roles in the occurrence, development, metastasis, and treatment of HCC, and provide new ideas and directions for the precision treatment of HCC.

## The DUB Family and Structure

It has been hypothesized that approximately 100 DUBs are encoded by the human genome. According to sequence and catalytic domain, DUBs are classified into 6 families: ubiquitin-specific proteases (USPs), ovarian tumor proteases (OTUs), ubiquitin C-terminal hydrolases (UCHs), Machado-Joseph domain-containing proteases (MJDs), the motif interacting with ubiquitin (MIU)-containing novel DUB family (MINDYs), and JAMM/MPN domain-associated Zn-depend metalloproteases (JAMMs)^16^.

**Figure 1:**
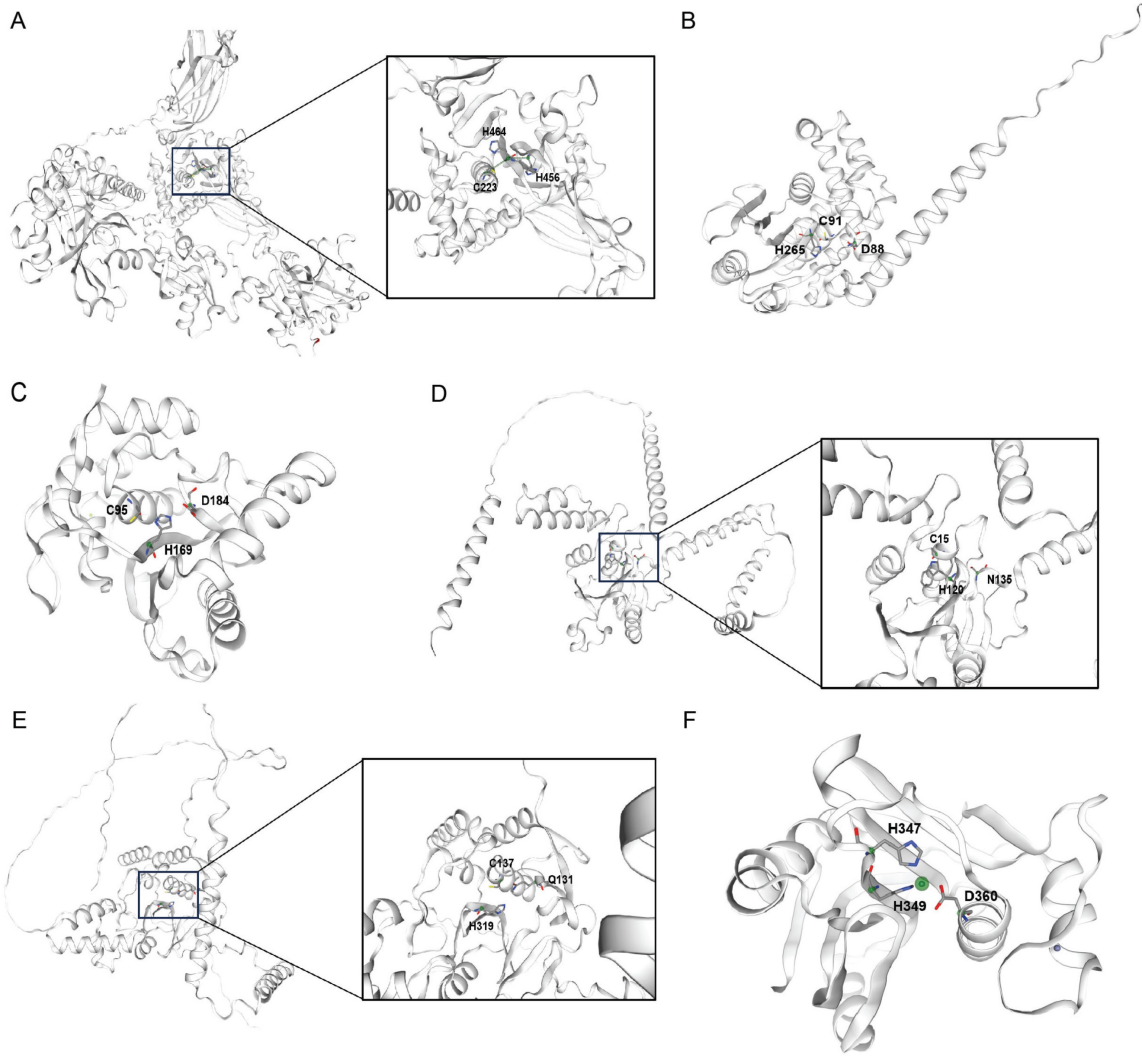
** Structural representations of DUB catalytic domains across six families.** Active site cysteine (for OTUs, USPs, UCHs, MJDs, MINDYs) or zinc (for JAMMs) is highlighted. Representative structures are shown for:(A) USP family (USP7, PDB: 1NB8) (B) OTU family (OTUB1, PDB: 2ZFY) (C) UCH family (UCH-L3, PDB: 1UCH) (D) MJD family (Ataxin-3, PDB: 1YZB) (E) MINDY family (MINDY1, PDB: 5JKN) (F) JAMM family (STAMBPL1, PDB: 2ZNR).

The USPs range from 50 to 300kDa, and their enzymatic functions are carried out by the thiol group of the central cysteine^17^. The catalytic region of a USP features a structure akin to that of papain, and this conserved domain allows the majority of USPs to cleave isopeptide bonds through a comparable process (Fig. 1A)^18^. USPs possess specialized cysteine protease domains that operate through distinct catalytic mechanisms; these regions are composed of three conserved subdomains, which are analogous to the anatomical components of the palm, thumb, and fingers of a human right hand, respectively^18^. The majority of USP family members consist of a central catalytic domain, along with additional N- and C-terminal extensions. These extensions can vary in composition, incorporating distinct domains or sequences that confer specialized functions^19^.

The OTU domain was initially discovered through bioinformatics analysis in a gene from ovarian tumors in Drosophila melanogaster^20^. Further studies revealed that certain members of the OTU family function as deubiquitinating enzymes, featuring potential catalytic cysteine and histidine residues^21^, ^22^. Almost all OTUs possess an OTU catalytic domain along with an ubiquitin interaction domain. The OTU core domain's structure consists of β-strands that are sandwiched between α-helical regions, and the α-helical regions are formed by an anterior α-helical region, a central β-sandwich region, and a posterior α-helical region^21^, ^23^. The OTUs' active site is established at the midpoint of the OTU's surface, precisely where the helical domain intersects with the β-strands domain (Fig. 1B)^24^. Although the overall structure of OTUs varies, the arrangement of catalytic residues is similar and conserved^25^.

The UCH subgroup comprises four members, which are UCH-L1, UCH-L3, UCH37, and BRCA1-associated protein-1. Each UCH featuring an N-terminal C12 peptidase domain that is shaped by a knotted peptide backbone, a C-terminal extension, and an unstructured loop that modulates substrate access to the catalytic site. The active site of a UCH typically consists of two parts: one is the central β-sheet structure, and the other is the α-helical structure surrounding the β-sheet. Furthermore, UCHs also contain an unstructured loop structure that limits the binding of substrates to the active site (Fig. 1C)^26^. In the absence of substrate, the active sites of UCHs are distorted and inactive. However, when bound to ubiquitin, the structures of UCHs active sites undergo changes, acquiring enzymatic activity, allowing the catalytic triad—composed of aspartic acid, histidine, and cysteine—to bind with the substrate^26^.

The MJD family encompasses several distinct deubiquitinating enzymes, including Ataxin-3, Ataxin-3L, JOSD1, and JOSD2, all of which contain a Josephin catalytic domain composed of approximately 180 amino acids^27^. The Josephin domain of MJD family proteins is responsible for their catalytic activity, while additional structural domains are involved in regulating their substrate specificity and subcellular localization (Fig. 1D)^27^.

MINDYs represent a relatively newly discovered family of deubiquitinating enzymes that contain an uncharacterized catalytic domain. Their crystal structure reveals a unique protein folding that shows no homology with any known deubiquitinating enzymes, but MINDYs still belong to the superfamily of cysteine proteases^28^. The catalytic core domain of MINDY-1 resembles a light bulb consisting of two subdomains, a central ''bulb'' subdomain that sits on a ''stalk'' subdomain, which resembles the base of the bulb (Fig. 1E)^28^.

Unlike other families of deubiquitinating enzymes, the JAMM family of deubiquitinating enzymes is a class of metalloproteases with a metal catalytic center, which typically contains a zinc ion that plays a key role in the catalytic reaction. JAMMs are typically composed of several distinct structural domains, including an MPN domain that is involved in metal ion binding and the catalytic reaction, as well as a JAMM domain that participates in substrate recognition and binding. JAMMs utilize a zinc ion in the metal catalytic center to polarize water molecules, which then attack the carbonyl carbon of the ubiquitin molecule, leading to the cleavage of the isopeptide bond between ubiquitin and the target protein (Fig. 1F).

## Function of the USP Family and Research Advances in Hepatocellular Carcinoma

The USP family is currently the most extensively studied family. This article will proceed to introduce the research progress of USPs in HCC.

### USP1

The best-characterized function of USP1 is as a regulator of several important steps in the DNA damage response^29^. In addition, recent evidence suggests that USP1 may also contribute to regulate differentiation in specific cellular contexts^30^. Current studies have found that USP1 is overexpressed in liver cancer tissue and associated with poor survival in HCC patients^31^, ^32^. And it has been proven that ML-323 (a USP1 inhibitor) inhibits the growth of HCC cells and induces G1 phase cell cycle arrest by regulating the expression of cell cycle proteins. Additionally, treatment of HCC cells with ML-323 results in the accumulation of ubiquitinated proteins, induces endoplasmic reticulum stress, and triggers Noxa-dependent apoptosis regulated by Activating Transcription Factor 4^31^. The Hippo signaling pathway has been proven to be a significant suppressive pathway in the development of HCC^33^. A study's DUB siRNA screening showed that USP1 is a key regulator of Hippo signaling activity. The mechanistic research of the article proves that USP1 interacts with the WW domain of TAZ, thereby enhancing the stability of TAZ through the inhibition of K11-linked polyubiquitination^32^. In addition, USP1 induces mitochondrial fission by enhancing phosphorylation of Drp1 at Ser616 via deubiquitination and stabilization of CDK5, thereby enhancing the metabolic reprogramming of HCC and affecting the occurrence and development of HCC^34^.

### USP2

The dysregulation of USP2 expression is closely related to the progression of HCC. Studies have found that compared with adjacent non-tumor tissue or normal liver tissue, the protein and mRNA levels of USP2 in HCC tissue are significantly altered^35^. Among the different isoforms of USP2, USP2b is dominant in normal liver tissue but is markedly down-regulated in HCC^36^. In contrast, USP2a is up-regulated in HCC, and its increased expression is positively correlated with poor patient prognosis^35^, ^37^. The data of Chongqing Medical University indicate that USP2a can promote HCC progression via deubiquitination and stabilization of RAB1A^37^. Non-alcoholic steatohepatitis (NASH) is also one of the causes of HCC. USP2 is also believed to be involved in lipid metabolism, further regulating the progression of HCC^38^.

### USP3

USP3 protein is upregulated in HCC tissues, but its upregulation level is not related to clinical pathological staging. Knocking down USP3 inhibits the growth of HCC cells and induces apoptosis in HCC cells. It also inhibits the growth of drug-resistant HCC cells. After USP3 knockdown, the levels and activity of β-catenin in HCC cells decrease and can be reversed by Wnt activators (lithium)^39^.

### USP4

A study indicated that Cyclophilin A (CypA) is an important molecule in USP4-mediated oncogenic activity in HCC. USP4 interacts with CypA and inhibits CypA degradation through deubiquitination in HCC cells, thereby enhancing the stability of CypA. Subsequently, the USP4/CypA complex activates the MAPK signaling pathway and prevents the phosphorylation of CrkII, thereby promoting the progression of HCC^40^. USP4 also interacts with TGFR-1 and deubiquitinates it, thereby activating the TGF-β signaling pathway and subsequently inducing epithelial-mesenchymal transition (EMT) in HCC cells^41^. Another study has elaborated on a different pathway affecting the EMT in HCC. LOXL3 interacts with the snail family transcriptional repressor 1 (Snail1), and Snail1 binds to the promoter of USP4, while USP4 deubiquitinates and stabilizes the expression of Snail1, thus forming a regulatory loop^42^.

### USP5

USP5 is overexpressed in highly malignant HCC and is positively correlated with the expression of SLUG. Downregulation of USP5 inhibits the deubiquitination of SLUG, thereby suppressing the proliferation, metastasis, and invasion of HCC cells, while the overexpression of USP5 promotes the stability of SLUG and EMT both in vitro and in vivo^43^. USP5-mediated deubiquitination of LSH suppresses ferroptosis in liver cancer cells by upregulating SLC7A11, thereby promoting the occurrence of HCC. Moreover, the USP5 inhibitor can inhibit the DUB activity of USP5 towards LSH, thereby suppressing the progression of HCC^44^. The P14(ARF) -P53 signaling was activated by USP5 knockdown in HCC cells. And Hpn induces apoptosis by inhibiting USP5 expression and activating the p14(ARF)-P53 signaling pathway^45^. CREB1/P300 serves as a key transcriptional regulator of METTL5, which controls the translation process of USP5, thereby regulating the ubiquitination of c-Myc and subsequently modulating the glycolytic metabolism in HCC^46^.

### USP7

USP7 expression in HCC tissues is significantly higher than in the surrounding non-tumor tissues, and it is associated with shorter overall survival and a higher cumulative recurrence rate of HCC^47^-^49^. USP7 modulates the Hippo pathway by regulating the ubiquitination status of Yes-associated protein (YAP). The transcriptional coactivator Yorkie, a key effector of the Hippo pathway, has its binding with USP7 diminished upon pathway activation, yet this activation promotes the phosphorylation of Yorkie^50^. Transmembrane protein 43 (TMEM43) plays a significant role in cancer, with a study revealing its high expression in HCC. Knockdown of TMEM43 has been shown to inhibit tumor progression. The research further discovered that USP7 regulates the ubiquitination levels of TMEM43 through deubiquitination, thereby affecting the progression of HCC^51^. Yin-yang-1 (YY1) is a DNA-binding protein with a zinc finger structure and is an important transcription factor that regulates the proliferation, migration, and EMT of tumor cells. USP7 can prevent the ubiquitin-dependent degradation of YY1, stabilizing the expression of YY1, thereby promoting the proliferation, migration, and EMT of HCC cells^52^. Mass spectrometry analysis revealed BTF3 as a potential substrate of USP7. Consequently, Hu and colleagues conducted a series of studies confirming the interaction between USP7 and BTF3, and that USP7 downregulates the ubiquitination levels of BTF3, thereby stabilizing BTF3 protein. Overexpression of BTF3 partially restored the inhibitory effects of USP7 depletion on the malignant phenotype and stemness characteristics of HCC cells^53^. USP7 can also form complexes with certain target proteins, thereby regulating downstream pathways. USP7 forms a complex with thyroid hormone receptor-interacting protein 12 (TRIP12), thereby stabilizing TRIP12. TRIP12, an E3 ubiquitin ligase, further induces the ubiquitination of constitutive p14(ARF) and promotes the progression of HCC^48^. TRIM27 is also an E3 ubiquitin ligase that is stabilized through binding to USP7. The USP7-TRIM27 complex regulates tumor progression in HCC by activating STAT3^54^. USP7 also has upstream regulatory factors. MiR-205 may negatively regulate the protein levels of USP7 in HCC cells by targeting the 3'-untranslated region (3'-UTR) of USP7, thereby modulating the p53 signaling pathway and cellular proliferation levels^47^. ENKUR inhibits the nuclear translocation of MYH9 by binding to β-catenin, reducing the expression of MYH9. The low expression of MYH9 suppresses the recruitment of the deubiquitinating enzyme USP7, promotes the degradation of c-Myc, and inhibits the proliferation, metastasis, and sorafenib resistance of HCC^55^. The results of qRT-PCR showed that METTL3 and USP7 are both highly expressed in HCC tissues and cell lines, and they are positively correlated. Further experiments indicated that METTL3 may regulate the expression of USP7 through m6A methylation, promoting the invasion, migration, and proliferation of HCC cells^56^. Another study demonstrated that flap endonuclease 1 (FEN1) can prevent the ubiquitination and degradation of mouse double minute 2 (MDM2) by recruiting USP7, thereby inactivating the p53 signaling pathway^57^. In addition to the specific USP7 inhibitor P22077, arsenic can also inhibit the expression of USP7, playing a role in inhibiting tumor development^58^, ^59^. However, arsenic is cytotoxic, and targeted delivery of arsenic drugs to liver tumors may be one of the research directions for the future.

### USP8

USP8 was initially discovered in breast cancer and is associated with the regulation of the epidermal growth factor receptor (EGFR)^60^. Subsequent studies have found that the upregulation or mutation of USP8 stabilizes numerous oncogenes or proto-oncogenes, thereby activating multiple signaling pathways, leading to cancer progression and survival^61^. Compared with normal liver tissue, USP8 is significantly upregulated in liver cancer tissue^62^-^64^. A study found that inhibiting USP8 can lead to the suppression of sensitive and resistant to doxorubicin HCC cell growth, as well as induce apoptosis. Meanwhile, the inhibition of USP8 resulted in a 90% reduction in the levels of various receptor tyrosine kinases (RTKs), including EGFR and c-Met^62^. Our research group conducted some studies and found that USP8 regulates ferroptosis in HCC. One of our studies indicated that USP8 stabilizes β-catenin through deubiquitination, thereby promoting the growth, invasion, tumor stem-like characteristics, and ferroptosis resistance of HCC^63^. Our other study found that targeting USP8 can inhibit the stability of O-GlcNAc transferase (OGT), subsequently suppressing the O-GlcNAcylation of SLC7A11, ultimately promoting ferroptosis in HCC^65^. Our research has also found that USP8 stabilizes OGT through deubiquitination, thereby promoting the progression of intrahepatic cholangiocarcinoma (ICC). At the same time, the absence of USP8 enhances the response of ICC to pemigatinib^64^.

However, another study has presented an opposing conclusion, suggesting that USP8 inhibits the progression of liver cancer by regulating TRAF6-mediated signaling to activate NF-κB and induce autophagy^66^. The researchers incorporated lipopolysaccharide (LPS), a key component of Gram-negative bacteria and potent TLR4 activator known to modulate the tumor immune microenvironment, in their investigation of USP8-mediated hepatocellular carcinoma regulation. This experimental design focusing on immune microenvironment mechanisms may account for the divergent conclusions observed in this study.

In tumor tissues of other organ systems (including lung, gastric, and pancreatic cancers), USP8 functions as a tumor suppressor. Targeted modulation of USP8 not only inhibits tumor progression but also enhances sensitivity to anti-tumor agents.

### USP9X

USP9X is highly expressed in liver cancer tissues and is an independent risk factor for poor prognosis. Knockdown of USP9X significantly inhibits the proliferation of HCC cells. Mechanistically, USP9X regulates the expression of β-catenin to promote HCC cell proliferation^67^. In HCC cell lines, miR-26b targets the 3'UTR of USP9X, thereby affecting EMT through the Smad4 and TGF-β signaling pathways^68^. In HCC, the expression level of miR-26b is reduced and negatively correlated with HCC grading^69^. Another study has shown that in HCC, miR-26b inhibits USP9X-mediated p53 deubiquitination, enhancing the sensitivity of HCC cells to doxorubicin^70^. Long non-coding LNC473 can recruit the deubiquitinating enzyme USP9X to inhibit the ubiquitination level of survivin, thereby increasing the expression of survivin, which in turn promotes the progression of HCC^71^.

### USP10

Current research has demonstrated that USP10 regulates the development of HCC, but the mechanisms are complex. Regarding its role as an oncogene or a tumor suppressor, some studies have reached contradictory conclusions. An article published by Dalian Medical University in 2018 stated that USP10 inhibits the progression of HCC by suppressing the activation of mTOR^72^. The team continued their research and found that in HCC, USP10 removes the ubiquitination of LKB1, thereby inhibiting the activation of mTOR^73^. The continuous activation of Smad4 and TGF-β is closely related to the metastasis of advanced HCC, while USP10 can maintain the protein levels of Smad4 and activate TGF-β signaling, thereby promoting the metastasis of HCC^74^. The small molecule inhibitor of USP10 can suppress the development of HCC by inhibiting the deubiquitination of YAP, thereby promoting the degradation of YAP and downregulating P53^75^. Growing evidence suggests that chronic stress, such as depression, is associated with poor prognosis in cancer patients, and the upregulation of PLAGL2 is related to chronic stress^76^-^78^. USP10 forms a signaling loop with PLAGL2, and stress-induced secretion of adrenaline activates this loop, thereby promoting the progression of HCC^79^.

### USP11

A study evaluated the expression of USP11 in 71 clinical samples of HCC by using immunohistochemical and analyzed the relationship between USP11 expression and clinical pathological characteristics and overall survival time. The study has found that the expression level of USP11 in tumor tissue is higher than that in non-tumor tissue, and is associated with vascular invasion, differentiation, number of tumors, recurrence rate, and shorter overall survival time^80^. The research team further discovered that USP11 interacts with nuclear factor 90 (NF90), promoting its deubiquitination, thereby stabilizing its function in HCC cells^81^. A study from Jinan University has reported that USP11 interacts with E2F1, maintaining the stability of E2F1 protein by deubiquitination, while E2F1 regulates the expression of USP11 at the transcriptional level, thus forming a positive feedback loop between E2F1/USP11 that promotes the proliferation and migration of HCC cells^82^. Another study demonstrated that USP11 promotes EMT and metastasis in HCC via eEF1A1/SP1/HGF dependent-EMT^83^. USP11 has been reported to regulate the metabolism of liver cancer cells. Elevated levels of USP11 have been detected in both in vitro models of hepatic steatosis and in public clinical data from patients with non-alcoholic fatty liver disease and HCC^84^. Under in vitro steatotic conditions, the absence of USP11 results in reduced lipid content and downregulation of genes involved in fatty acid metabolism^84^. Furthermore, USP11 modulates glucose metabolism in HCC through the HIF1α/LDHA axis, thereby affecting proliferation and metastasis^85^.

### USP12

Compared with adjacent normal tissues, USP12 is specifically upregulated in liver cancer tissues. After knocking down USP12, the cell cycle protein-dependent kinase 1/cyclinB1 axis was activated, inducing G2/M arrest, and triggering apoptosis through the p38/ MAPK pathway^86^. Another study found that USP12 can remove the ubiquitination of c-Myc, thereby regulating the progression of HCC^87^.

### USP13

Nanjing medical university investigated the function of USP13 in HCC, they found that USP13 was significantly upregulated in both of primary HCC tumor tissues and cell lines. And mechanism studies indicated that knocking down USP13 can significantly downregulate the expression levels of c-Myc and overexpression of c-Myc could significantly attenuate the effects of shUSP13 on HCC cell growth inhibition^88^. However, the study did not clarify whether USP13 regulates c-myc through the deubiquitination pathway or other pathways. The activation of the Toll-like receptor 4/Myeloid differentiation primary response gene 88/Nuclear factor-kappa B (TLR4/MyD88/NF-κB) pathway is involved in the occurrence and development of HCC. Moreover, studies have shown that TLR4 in melanoma is regulated by the ubiquitin-proteasome system. Gao et al. found that USP13 inhibits ubiquitin-mediated TLR4 degradation, thereby enhancing the proliferation, EMT, migration, and invasion of HCC^89^.

### USP14

USP14 exhibits significantly higher expression in tumor tissues of HCC patients compared to para-cancerous and normal liver tissues. Moreover, patients with higher USP14 expression have a markedly poorer prognosis after surgery than those with lower levels of USP14 expression^90^. Researchers have begun to explore the mechanisms by which USP14 influences the occurrence and development of HCC. A study from Wuhan University has found that the selective USP14 inhibitor, b-AP15, induces cytotoxicity in HCC cells by increasing endoplasmic reticulum (ER) stress and inhibiting the Wnt/Notch1 signaling pathway^91^. This suggests that USP14 may affect the tumor activity of HCC by activating the Wnt/Notch1 signaling pathway. China Medical University demonstrated that USP14 is involved in the maintenance of HIF1α stability to activate HIF1α-induced transactivation via its deubiquitinase activity. And USP14 depletion or its specific inhibitor treatment decreased cell proliferation, invasion, migration, and Vascular Mimicry formation even under hypoxia conditions in HCC cell lines^92^. Another study of Affiliated Hospital of Nantong University indicated that knocking down USP14 significantly inhibits the proliferation, invasion, and metastasis of liver cancer cells, promotes apoptosis, and simultaneously suppresses the expression of hexokinase 2 (HK2). Further experimental research has found that USP14 affects EMT through the HK2/AKT/P62 axis. At the same time, since HK2 is a downstream target of USP14, USP14 can mediate glucose metabolism in HCC cells^93^. USP14 also plays a significant role in the resistance mechanism of HCC to lenvatinib; silencing USP14 can markedly reduce lenvatinib resistance both in vitro and in vivo. The specific mechanism involves USP14 removing the ubiquitination of Calcium and Integrin-Binding Protein 1 (CIB1), thereby promoting the P21-activated kinase 1 (PAK1)-ERK1/2 signaling axis^94^.

### USP15

Current literature indicates that USP15 promotes the progression of HCC and is associated with the prognosis of HCC patients. A system biology analysis suggests that USP15 may suppress tumorigenesis in HCC by regulating gene expression, cell cycle, and signal transduction pathways involved in DNA repair^95^. After silencing the expression of USP15 in HCC cells, cell proliferation is inhibited and apoptosis is induced^96^. However, there is no further mechanistic research.

### USP16

Hepatitis B virus X protein (HBx) has been shown to accelerate HCC progression by promoting tumor growth and metastasis^97^-^99^. Carboxyl-terminal truncated HBx (Ct-HBx) proteins are frequently present in HCC tumour tissues, but not in non-tumorous tissues^100^, ^101^. The expression of USP16 is significantly suppressed by Ct-HBX, thereby inhibiting tumor proliferation. Conversely, ectopic expression of USP16 suppresses the tumor-promoting activity of Ct-HBx^102^.

### USP18

Currently, only one article has elaborated on the role of USP18 in HCC. The article points out that the expression of USP18 in HCC tissue is higher than that in adjacent non-cancerous tissue, and it is even more highly expressed in HCC associated with hepatitis B virus infection. Moreover, it may promote the growth of HCC cells by stabilizing the BCL2L2 protein^103^.

### USP19

USP19 is upregulated in HCC tissues and is associated with poor prognosis. In vivo and in vitro experiments have also demonstrated that USP19 promotes the proliferation and migration of HCC. USP19 is a specific deubiquitinating enzyme for YAP, and knockdown of USP19 can reduce the expression of YAP protein and its target genes^104^.

### USP20

Our research group has identified that USP20 plays a role in inhibiting ferroptosis in HCC cells and enhancing their resistance to oxaliplatin. This effect is mediated by USP20's ability to remove K48-linked polyubiquitination at lysine residues K30 and K37 of the SLC7A11 protein, thereby stabilizing SLC7A11. The stabilization of SLC7A11 subsequently suppresses ferroptosis, which contributes to the development of oxaliplatin resistance. And the phosphorylation of USP20 at Ser132 and Ser368 requires the activation of ATR induced by DNA damage^105^.

### USP21

Li's team conducted an analysis of multiple HCC datasets and discovered that the USP21 gene, which encodes a member of the ubiquitin-specific protease family, is highly amplified and overexpressed in HCC. This upregulation is significantly associated with poor clinical prognosis. Inhibition of USP21 in HCC cell lines was found to reduce tumorigenic properties, while ectopic expression enhanced oncogenicity. Further mechanistic studies revealed that USP21 stabilizes MEK2 by removing its ubiquitination, thereby activating the ERK signaling pathway^106^. Circular RNAs (circRNAs) play a pivotal role in the oncogenesis of HCC, and the expression of USP21 is regulated by hsa_circ_0039053. Studies have shown that hsa_circ_0039053 can interact with miR-637 to promote the expression of USP21, thereby facilitating the progression of HCC^107^.

### USP22

A study of Ling found that USP22 promotes hypoxia-induced stemness and glycolysis in HCC cells by deubiquitination and stabilization of HIF1α. Moreover, USP22 and HIF1α formed a positive feedback loop and facilitating the development of HCC^108^. Researchers at the First People's Hospital of Hangzhou have identified another positive feedback loop involving USP22. In this loop, USP22 promotes the activation of mammalian target of rapamycin complex 1 (mTORC1) by deubiquitinating FK506-binding protein 12 (FKBP12), and the activated mTORC1 further stabilizes USP22 by inhibiting autophagic degradation. This discovery highlights the complex regulatory network of USP22 in the context of HCC and suggests that targeting this feedback loop could be a potential therapeutic strategy for HCC^109^. Elevated de novo lipogenesis is considered a crucial factor in the development of HCC^38^, and USP22 has been shown to promote the de novo synthesis of fatty acids and HCC tumorigenesis. The specific mechanism involves USP22 stabilizing peroxisome proliferator-activated receptor gamma (PPARγ) through deubiquitination, which in turn increases the expression of acetyl-CoA carboxylase (ACC) and ATP citrate lyase (ACLY)^110^. USP22 has been implicated in the regulation of the immunological microenvironment in HCC. The specific mechanism involves PRDM1 enhancing the transcription of USP22, which then reduces the degradation of SPI1 protein through deubiquitination, thereby enhancing the transcription of Programmed Cell Death Ligand 1 (PDL1). This ultimately leads to the exhaustion of infiltrating CD8+ T cells^111^.

### USP24

The function of USP24 in tumors is intricate, with various studies presenting contrasting views on whether USP24 serves as a promoter or suppressor of cancer. In the context of neuroblastoma, a deficiency in USP24 is noted, and the overexpression of USP24 has been demonstrated to suppress the proliferation of neuroblastoma cells^112^. Conversely, in lung and gastric cancers, USP24 behaves as an oncogenic factor^113^, ^114^. Similar to these findings, research on HCC has also produced inconsistent conclusions. Zhou's research discovered that USP24 binds to Tumor Necrosis Factor Receptor-Associated Factor 2 (TRAF2), inhibiting its degradation and thereby promoting the accumulation of TRAF2. The upregulation of TRAF2 activates the AKT/NF-κB signaling pathway, which enhances the survival of HCC cells. Additionally, USP24 expression is positively correlated with the expression of PDL1 in HCC^115^. Hu et al. discovered that in sorafenib-resistant HCC cells, miR-21-5p is upregulated, and USP24 is a downstream target of miR-21-5p. Further mechanistic exploration revealed that the upregulation of miR-21-5p increases USP24, which in turn removes the ubiquitination level of SIRT7 through USP24, thereby inhibiting cellular autophagy^116^. Researchers at Zhengzhou University believe that USP24 is a tumor suppressor. They have discovered that USP24 can stabilize Beclin1, one of the key regulators of autophagy, thereby promoting tumor cell autophagy and ferroptosis, and increasing the susceptibility of HCC to sorafenib^117^.

### USP25

The USP25 protein plays a crucial role in the development of various types of cancer. For instance, in pancreatic cancer, USP25 stabilizes HIF1α through deubiquitination, thereby promoting glycolysis in cancer cells and consequently driving tumor progression^118^. In tissues and cell lines of HCC, USP25 is also highly expressed. Research has confirmed that in HCC, USP25 interacts with TRIM21 to regulate EMT and the Wnt/β-catenin signaling pathway. These findings reveal the significant role of USP25 in the development of liver cancer and may provide new targets for future therapeutic strategies^119^.

### USP26

USP26 plays a regulatory role in the development and progression of colorectal, cervical, breast, and liver cancers. In HBV-positive HCC, USP26 is strongly induced, and its levels are associated with poor prognosis. Mechanistically, the HBV-encoded protein HBx binds to the promoter and induces the production of USP26, which in turn stabilizes SIRT1 through deubiquitination, thereby promoting cell proliferation, inhibiting apoptosis, and accelerating the occurrence of HCC^120^.

### USP27

Cyclin E is frequently dysregulated in tumor cells and this may contribute to the development of various types of cancers^121^-^124^, and USP27 promoted Cyclin E stability by negatively regulating its ubiquitination. In addition, suppression of USP27 expression results in the inhibition of the growth, migration, and invasion of hepatocellular carcinoma, and the absence of USP27 makes Hep3B cells sensitive to 5-FU-induced apoptosis, indicating that USP27 plays a role in the drug resistance of HCC^125^. USP27 can also remove the ubiquitination of the histone methyltransferase SETD3, thereby enhancing its stability and promoting the growth of tumor cells^126^.

### USP28

USP28 is overexpressed in the majority of tumors and holds prognostic value across various cancer types. Moreover, there is a significant correlation between USP28 and immune modulatory factors, clinical staging, checkpoint inhibitor responses, MSI (microsatellite instability), TMB (tumor mutational burden), CNV (copy number variation), MMR (mismatch repair) defects, and DNA methylation^127^. Among these, patients with liver cancer and high expression of USP28 generally have a poorer prognosis^128^. In HCC, it has been reported in literature that USP28 regulates tumor progression by removing the ubiquitination of c-Myc^128^, ^129^. USP28 is regulated by microRNAs and circular RNAs; miR-363-3p and miR-216b can inhibit the expression of USP28, while circMED27 upregulates USP28^128^-^130^. However, no further targets of USP28 have been identified in HCC.

### USP29

Aberrant expression of HIF1α and increased aerobic glycolysis metabolism are drivers of resistance to Sorafenib^131^, ^132^. USP29 is a key deubiquitinating enzyme of HIF1α, which directly deubiquitinates and stabilizes HIF1α, thereby promoting its transcriptional activity. HK2 is one of the transcriptional targets of HIF1α and is also a key enzyme in aerobic glycolysis. Knocking down USP29 reduces the level of aerobic glycolysis in sorafenib-resistant HCC cells, restoring their sensitivity to sorafenib^133^.

### USP30

The GNPAT, which encodes the enzyme glyceronephosphate O-acyltransferase, exhibits amplification, upregulation, and a strong correlation with poor clinical outcomes in patients with HCC. USP30 is recruited by GNPAT, which encodes the enzyme glyceronephosphate O-acyltransferase. Subsequently, USP30 deubiquitylates and stabilizes dynamin-related protein 1 (DRP1), thereby playing a crucial role in the regulation of mitochondrial morphology, lipid metabolism, and the initiation of HCC^134^. USP30 is prominently expressed in HCC that develops in mice subjected to a high-fat diet^135^. This indicates that USP30 is also involved in the regulation of lipid metabolism in HCC cells. IKKβ phosphorylates and stabilizes USP30, which in turn promotes the deubiquitination of ACLY and fatty acid synthase (FASN) by USP30. IKKβ also directly phosphorylates ACLY, facilitating the interaction between USP30 and ACLY, as well as the deubiquitination of the latter^135^.

### USP32

Several studies have found that USP32 can regulate the growth and development of different tumors through the catalytic activity of deubiquitinating enzymes. Analysis of the TCGA database has yielded the expression patterns of USP32 in various malignant tumors. Among them, six types of cancer, including cholangiocarcinoma, esophageal cancer, head and neck squamous cell carcinoma, hepatocellular carcinoma, and gastric adenocarcinoma, show elevated USP32 mRNA expression levels^136^. Xiu et al. conducted CCK8, migration assays, and clonogenic assays on HCC cell lines and established a nude mouse model of HCC to verify the carcinogenic role of USP32. The experiments demonstrated that the knockdown of USP32 can inhibit the proliferation, colony formation, and migration of HCC cells, as well as suppress tumor growth in vivo^137^. In gastric cancer, USP32 regulates the expression of SMAD2 in a ubiquitin proteasome-dependent manner^138^. Additionally, USP32 promotes the progression of non-small cell lung cancer by deubiquitinating BAG3 and activating the RAF-MEK-ERK signaling pathway^139^. However, the specific mechanisms by which USP32 regulates the development of HCC have not been further explored.

### USP33

Gan et al. first discovered the abnormal increase in USP33 expression in HCC tissues, and USP33 may serve as a prognostic biomarker for HCC patients. Furthermore, as a deubiquitinating enzyme for SP1, USP33 participates in the invasion and metastasis of HCC by activating the SP1/c-Met axis^140^.

### USP35

Immunohistochemical analysis comparing the expression of USP35 in HCC tumors and peritumoral tissues revealed a significant upregulation of USP35 in tumor tissues. Prognostic statistical analysis indicated that HCC patients with higher USP35 expression had significantly shorter overall survival times^141^. Mechanistically, USP35 promotes the development of HCC by deubiquitinating and stabilizing PKM2 and ABHD17C proteins, while also activating the PI3K/AKT signaling pathway^141^, ^142^.

### USP39

USP39 plays a regulatory role in the proliferation of HCC cells. Upon USP39 knockdown, cell proliferation is significantly suppressed, and apoptosis is induced^143^, ^144^. A study has observed that USP39 stabilizes the protein SP1 by promoting its deubiquitination, and USP39 facilitates the proliferation of HCC cells in an SP1-dependent manner^145^. Zinc-finger E-box-binding homeobox 1 (ZEB1) and β-catenin are associated with the proliferation and metastasis of HCC, and their protein levels are regulated by the ubiquitination system. Specifically, the E3 ubiquitin ligase TRIM26 suppresses HCC by ubiquitinating ZEB1 and β-catenin, while USP39 removes the ubiquitination. Additionally, USP39 inhibits the maturation and splicing of TRIM26 pre-mRNA, thereby further increasing ZEB1 and β-catenin levels^146^, ^147^. Knockdown of USP39 leads to the downregulation of the transcription factor Forkhead box M1 (FoxM1). The expression of FoxM1 target genes, including Polo-like kinase 1, Cyclin B1, and Centromere protein A, also decreases following USP39 knockout. These results suggest that USP39 may regulate the progression of HCC by modulating the pre-mRNA splicing of FoxM1^148^. A study has demonstrated that USP39 can be acetylated by the histone acetyltransferase MYST1, which leads to its degradation by the proteasome. Conversely, the lysine deacetylase sirtuin 7 (SIRT7) removes the acetylation of USP39, promoting its stability and consequently accelerating the proliferation and tumorigenesis of HCC cells both in vitro and in vivo^149^. USP39 is recruited by dynein axonemal assembly factor 5 (DNAAF5), and stabilize phosphofructokinase L (PFKL) through the deubiquitination pathway, thereby promoting HCC proliferation and resistance to sorafenib^150^.

### USP40

USP40 is upregulated in HCC tissues and predicts poor prognosis for HCC patients. Knockdown of USP40 inhibits the proliferation, migration, and stemness of HCC cells. Mechanistically, USP40 interacts with Claudin1 and suppresses its polyubiquitination, thereby stabilizing Claudin1 protein^151^. YAP is a crucial driver of HCC progression, and the ubiquitin-proteasome system governs its levels. USP40 interacts with YAP to remove the lysine 48 (K48)-linked polyubiquitination at K252 and K315 sites, thereby maintaining YAP stability. In turn, YAP transcriptionally activates the expression of USP40 in HCC cells. This reciprocal regulation forms a positive feedback loop between USP40 and YAP, which contributes to the progression of HCC^152^.

### USP44

In various types of cancer, the role of USP44 in tumorigenesis and development varies. In breast and prostate cancer, USP44 acts as an oncogenic factor. In contrast, in pancreatic cancer, thyroid cancer, colorectal cancer, and liver cancer, USP44 inhibits tumor progression. The expression of USP44 is diminished in HCC tissues, and clinical pathological analysis further indicates that low levels of USP44 expression are associated with poorer prognosis and advanced tumor stages in HCC patients^153^. A study investigating the mechanism by which USP44 suppresses tumors in HCC found that USP44 inhibits PDL1 expression by stabilizing Itch, which downregulates the Hedgehog (Hh) signaling pathway. Additionally, the Gli1 inhibitor GANT61 was found to synergize with anti-PDL1 therapy, suggesting a potential combined therapeutic approach for HCC treatment^154^.

### USP46

USP46 is found to be downregulated in HCC tissues, and low levels of USP46 are correlated with a poor prognosis for HCC patients. USP46 stabilizes MST1 protein by directly binding to it and reducing its ubiquitination, and MST1 antagonizes the expression of YAP, thereby inhibiting the development of HCC^155^. A series of experiments from Nanchang University have demonstrated that USP46 is a target gene of miR-27a-3p. MiR-27a-3p promotes the proliferation of cancer cells and accelerates the occurrence and development of HCC by inhibiting the expression of USP46^156^.

### USP48

Pyroptosis is a form of programmed cell death characterized by the activation of inflammatory caspases and the cleavage of gasdermin proteins. It can suppress tumor development and induce antitumor immunity, making the activation of pyroptosis a potential therapeutic strategy for cancer. USP48, by stabilizing Gasdermin E (GSDME), promotes pyroptosis and enhances the therapeutic efficacy of programmed cell death protein 1 inhibitors in mouse tumor models^157^. Additionally, in mouse liver tumor induced by diethylnitrosamine and human HCC, the expression of USP48 is downregulated. In HCC, USP48 stabilizes SIRT6 by deubiquitination, which hinders metabolic reprogramming and thus inhibits HCC tumorigenesis. Furthermore, the m6A modification induced by methyltransferase-like 14 (Mettl14) is involved in the regulation of USP48 in HCC by maintaining the stability of USP48 mRNA^158^.

### USP49

USP49 is a downstream molecule of HLNC1, which is a long non-coding RNA. HLNC1 interacts with USP49 and significantly destabilizes it, thereby promoting the viability and migration of HCC cells^159^. USP49 is also a tumor suppressor in pancreatic cancer^160^. However, in gastric cancer, USP49 stabilizes YAP1 through deubiquitination, and YAP1 in turn promotes the transcription of USP49, forming a positive feedback loop that drives the malignant progression of gastric cancer^161^.

### USP53

The antitumor effect of USP53 has been confirmed in lung and breast cancer, and USP53 expression is downregulated in HCC tissues as well as HCC cell lines. Furthermore, the ectopic expression of USP53 inhibits the proliferation, migration, and invasion of HCC cells and induces apoptosis in HCC cells. Immunoprecipitation (CO-IP) assays and mass spectrometry analysis identified cytochrome c (CYCS) as a target protein of USP53, and USP53 stabilizes CYCS to induce apoptosis in HCC cells^162^.

### CYLD

CYLD (Cylindromatosis) contains a USP catalytic domain and belongs to the USP family, capable of cleaving K63 and M1 linked polyubiquitin chains. Initially associated with the rare disease familial cylindromatosis, subsequent research has found that CYLD is involved in the regulation of the occurrence and development of various tumors. Compared to the surrounding non-malignant tissues, the protein and mRNA levels of CYLD in HCC are both lower, and it is believed that downregulating CYLD in HCC cell lines increases the resistance of HCC cells to treatment with doxorubicin, 5-fluorouracil, and cisplatin. Further findings show that the downregulation of CYLD reduces the apoptosis induced by tumor necrosis factor-α, while also leading to the degradation of the NF-κB inhibitor IκB-α, thereby enhancing NF-κB activity in HCC cells^163^. And Pannem's research found that CYLD negatively controls the expression of c-MYC in HCC through the JNK-dependent signaling pathway, thereby regulating the proliferation of HCC cells^164^. Existing research suggests that multiple microRNAs regulate the expression of CYLD, such as miR-362^165^, miR-501^166^, ^167^, and miR-922^168^. Lu's research team found in the liver cancer cell line HepG2 that SOX2 increases the expression of EGFR, and EGFR upregulates miR-222-5p, leading to the downregulation of CYLD^169^.

## Function of the OTU Deubiquitinase Family and Research Advances in Hepatocellular Carcinoma

Current research indicates that OTU deubiquitinating family members, including OTUB1, OTUD3, OTUD4, OTUD5, and TRABID, are associated with HCC. This article will further elaborate on the relationship between these OTUs and the occurrence and progression of HCC.

### OTUB1

Chen's research team found that the expression level of OTUB1 is significantly correlated with clinical pathological parameters such as TNM staging, histological staging, metastasis/recurrence by analyzing 115 clinical HCC tissue samples. Survival analysis showed that patients with high expression of OTUB1 had a lower overall survival time compared to those with low expression of OTUB1. In vitro experiments revealed that knocking down OTUB1 inhibited the proliferation, migration, and invasion capabilities of HCC cells^170^. Overexpression of OTUB1 in HCC could be a novel, effective, and supplementary biomarker for HCC. However, the specific mechanisms by which OTUB1 regulates the progression of HCC still require further investigation.

### OTUD3

OTUD3 is also significantly overexpressed in HCC. Furthermore, mass spectrometry analysis revealed that α-actinin 4 (ACTN4) is a downstream target of OTUD3, and the protein level of ACTN4 is significantly correlated with the expression of OTUD3. Further studies found that OTUD3 directly binds to ACTN4, leading to its deubiquitination and stabilization. Both in vitro and in vivo experiments confirmed that ACTN4 is crucial for OTUD3-mediated HCC proliferation and metastasis^171^.

Another article, however, revealed that OTUD3 acts as a tumor suppressor in hepatocellular carcinoma while simultaneously enhancing the sensitivity of liver cancer cells to sorafenib treatment. The study found that OTUD3 is a deubiquitinating enzyme for the eukaryotic initiation factor 2α (eIF2α), which can antagonize the integrated stress response (ISR) and inhibit liver cancer^172^. ISR is a response activated in response to intrinsic and extrinsic stimuli and plays a role in tumor progression and drug resistance^173^-^175^. Moreover, the activation of ISR due to reduced OTUD3 expression confers resistance of liver cancer cells to sorafenib, and the combined use of the ISR inhibitor ISRIB significantly enhances the sensitivity of liver cancer cells to sorafenib^172^.

OTUD3, a deubiquitinating enzyme, demonstrates functional pleiotropy in tumorigenesis through its regulation of multiple substrate targets. While its roles in HCC have been partially characterized, accumulating evidence reveals that OTUD3 can function as either a tumor suppressor or oncoprotein in a context-dependent manner across various malignancies, including lung, breast, and colorectal cancers. These seemingly contradictory findings underscore the complexity of OTUD3's mechanistic involvement in HCC pathogenesis and highlight the need for further investigation to fully elucidate its molecular functions.

### OTUD4

OTUD4 mRNA expression is significantly downregulated in HCC tissues and is associated with poor prognosis. Through gene set enrichment analysis (GSEA), OTUD4 is related to apoptosis signaling pathways and the AKT signaling pathway. Therefore, the overexpression of OTUD4 may inhibit the proliferation, migration, and invasion of HCC cells by suppressing the AKT signaling pathway^176^. The specific mechanisms by which OTUD4 regulates the development of HCC cells remain unclear and warrant further investigation.

### OTUD5

The research team from Xi'an Jiaotong University found that OTUD5 is significantly upregulated in HCC tissues, and high levels of OTUD5 are also detected in most HCC cell lines. Analysis of TCGA data showed that high expression of OTUD5 indicates a poorer overall survival in HCC patients. Mass spectrometry analysis revealed that solute carrier family 38 member 1 (SLC38A1) is a candidate downstream target protein of OTUD5. Further research results indicate that OTUD5 stabilizes SLC38A1 by preventing its ubiquitin-mediated proteasomal degradation, thereby promoting the proliferation of HCC cells^177^.

### OTUD7B

Recent studies have revealed that the deubiquitinase OTUD7B, which is significantly downregulated in HCC, directly binds to and removes lysine-linked polyubiquitin chains from both wild-type and mutant p53, thereby inhibiting its proteasomal degradation and enhancing p53 protein stability. Functional experiments demonstrated that OTUD7B suppresses HCC cell and xenograft growth by activating the p53-dependent mitochondrial apoptosis pathway (inducing PUMA/BAX expression), an effect that is abolished upon p53 depletion. Notably, OTUD7B and p53 form a bidirectional regulatory loop—OTUD7B expression is itself transcriptionally repressed by p53. Clinical data analysis showed a positive correlation between OTUD7B and p53 protein levels in HCC tissues, suggesting that the OTUD7B-p53 axis may represent a potential therapeutic target for HCC^178^.

### TRABID

TRAF-binding domain-containing protein (TRABID) also is a member of the OTU deubiquitinase family. Proteomic analysis based on mass spectrometry has found that TRABID associates with damaged DNAAF5 (DDB2) in HepG2 cells. TRABID causes deubiquitination of DDB2, and the proliferation of HCC cells mediated by TRABID is attenuated by DDB2 knockdown^179^.

## Function of the UCH Family and Research Advances in Hepatocellular Carcinoma

Despite growing evidence suggesting that UCH enzymes are closely related to human malignant tumors, there have been few studies on the role of UCH enzymes in HCC. Moreover, there are currently no studies that demonstrate the impact of UCH-L1 and UCH-L3 on the development of HCC.

### UCH37

Zhongshan Hospital of Fudan University performed a functional proteomic analysis to screen UCH37-interacting proteins in hepatocellular carcinoma, and glucose-regulated protein 78 (GRP78) was identified as one interacting with UCH37^180^. GRP78 may be a target protein of UCH37, but there is a lack of research on its specific mechanisms. Zhongshan Hospital has conducted further research on UCH37 and found that UCH37 is overexpressed in HCC, making it an important indicator for predicting time to recurrence. Concurrently, in vitro experiments have confirmed that UCH37 can promote cell migration and invasion in HCC cell lines by interacting with and deubiquitinating the RNA splicing factor PRP19^181^.

### BRCA1-associated protein-1 (BAP1)

Compared with the adjacent non-tumor tissues, the levels of BAP1 mRNA and protein in HCC are significantly downregulated. Low expression of BAP1 is positively correlated with aggressive tumor phenotypes, and is independently associated with poorer recurrence-free survival and overall survival following curative hepatectomy. Wild-type BAP1, but not mutant BAP1, significantly inhibits the proliferation, invasion, EMT of HCC cells in vitro, and tumor progression and metastasis in vivo. Mechanistically, BAP1 interacts with PTEN and stabilizes PTEN through deubiquitination, and further negatively regulates HCC cell EMT by inactivating the AKT/GSK-3β/Snail pathway. However, these tumor-suppressive effects of BAP1 are abolished by inactivating mutations^182^.

## Function of the MJD Family and Research Advances in Hepatocellular Carcinoma

Machado-Joseph disease, also referred to as spinocerebellar ataxia type 3, is a neurological disorder that is widely recognized as the most common form of spinocerebellar ataxia. MJDs are closely associated with such neurological conditions^183^.

As a member of MJDs and a therapeutic target in neurodegenerative disease, Ataxin-3 has been well characterized in the progression of MachadoJoseph disease^184^. As for other members of MJD such as Ataxin-3L, JOSD1, and JOSD2, there is no evidence to suggest their role in MJD. However, a growing body of evidence indicates that Ataxin-3, Ataxin-3L, JOSD1, and JOSD2 are also associated with cancer progression. Recent studies have indicated that non-small cell lung cancer is associated with Ataxin-3, testicular cancer and human colorectal cancer are also related to Ataxin-3^185^-^187^. Buss R et al. found that Ataxin-3L promotes the migration of NSCLC cells, and high levels of are associated with lower overall survival rates in breast cancer patients^188^, ^189^. But the mechanism is not yet clear. JOSD1 is frequently overexpressed in lung adenocarcinoma, head and neck squamous cell carcinoma, colon cancer, uterine cancer, melanoma, ovarian cancer, and bladder cancer^189^-^193^. Amplification of JOSD2 is more common in tumors such as uterine cancer, cholangiocarcinoma, lung cancer, adrenocortical carcinoma, diffuse large B-cell lymphoma of the lymphoid type, bladder cancer, breast cancer, gastric cancer, and pancreatic cancer^189^, ^194^, ^195^. Zhou et al. conducted a study and found that the expression levels of Ataxin-3, JOSD1, and JOSD2 were significantly elevated in HCC tissues and cell lines. These elevated expressions correlated with histological grading, specimen types, TP53 mutations, lymph node metastasis, gender, and age of HCC patients^196^. A study conducted by Fujian Medical University has delved into the mechanism by which JOSD2 regulates HCC. The findings indicate that JOSD2 interacts with and reduces the ubiquitination levels of catenin β 1 (CTNNB1), thereby enhancing the transmission of the Wnt pathway^197^.

## Function of the MINDYs and Research Advances in Hepatocellular Carcinoma

As a newly identified family of deubiquitinating enzymes, the MIDNYs have not been extensively studied and applied in cancer research. Current research indicates that MINDY1 is associated with breast cancer, bladder cancer, and HCC, and promotes their progression^198^-^201^.

In human breast cancer tissues, there is a positive correlation between estrogen receptor α (ERα) and MINDY1 protein levels, and high expression of MINDY1 is associated with poor prognosis. Further mechanistic research has found that MINDY1 mediates the deubiquitination of ERα and increases its stability in a deubiquitination activity-dependent manner^198^. In bladder cancer, MINDY1 has been shown to interact with YAP in a deubiquitination activity-dependent manner, deubiquitinating and stabilizing YAP^199^. Xia et al. have discovered that the expression level of MINDY1 in HCC tissues is higher than that in adjacent tumor tissues and is closely related to the progression of the tumor. High expression of MINDY1 is an independent risk factor for poor prognosis in HCC patients^201^. Song and colleagues have found that MINDY1 inhibits the malignant progression of HCC by suppressing the ubiquitination of PDL1 and mediating immune escape^200^.

## Function of the JAMM Family and Research Advances in Hepatocellular Carcinoma

Members of the JAMM deubiquitinating enzyme family have been implicated in various cancers. Specifically, PSMD14 has been associated with glioblastoma, where it promotes tumorigenesis by deubiquitinating and stabilizing β-catenin, a key component of the Wnt signaling pathway^202^. And PSMD14 also is a novel prognostic marker and therapeutic target in osteosarcoma^203^. STAMBP has been linked to breast cancer and melanoma, potentially contributing to the progression of these cancers through its deubiquitinating activities^204^-^206^. Mutations in BRCC3 have been correlated with myeloid neoplasms, suggesting a role in the development of blood-related cancers^207^. Lastly, STAMBPL1 has been identified as being associated with HCC. The clinical results of the study show that STAMBPL1 is significantly increased in tumor tissues of HCC patients, and its expression is closely related to tumor size and TNM staging. Cells with overexpression of STAMBPL1 exhibit a marked enhancement in proliferation both in vitro and in vivo, while STAMBPL1 knockdown demonstrates the completely opposite effect. Mechanistically, STAMBPL1 activates the Wnt/β-catenin pathway, increasing the expression of downstream oncogenes. In addition to this, STAMBPL1 is transcriptionally regulated by Sterol Regulatory Element-Binding Protein 1 (SREBP1), and overexpression of STAMBPL1 increases lipid accumulation in HCC cells and xenografted tumors. These findings suggest that STAMBPL1 plays a role in the malignant behavior of HCC cells by modulating the Wnt/β-catenin pathway and lipid metabolism^208^.

## Conclusions and Future Perspectives

This article introduces and analyzes some DUBs related to HCC, indicating that DUBs play a unique regulatory role in the occurrence and development of HCC. The development of targeted drugs has become an important treatment modality for patients with high expression of deubiquitinating enzymes in HCC, and some molecular targeted drugs and small molecule inhibitors related to ubiquitination and deubiquitination enzymes have also shown therapeutic effects in clinical treatment^209^-^211^.

Due to the involvement of multiple pathways and targets, the regulatory mechanisms of deubiquitinating enzymes in HCC are complex. Most DUBs associated with HCC tend to promote tumor activity, However, OTUD4 and BAP1 have been shown to exert inhibitory effects on tumorigenesis. The roles of USP8 and OTUD3 in HCC are less clear, with some studies yielding contradictory results regarding their oncogenic or tumor-suppressive functions. This suggests that the mechanisms by which USP8 and OTUD3 regulate HCC are more intricate and require further elucidation.

The regulatory mechanisms involving DUBs in HCC are extremely complex, with DUBs possessing multiple downstream targets. To provide a more intuitive understanding of the process by which DUBs regulate HCC, this article offers a table and a schematic diagram to illustrate the complex processes involved in the occurrence and development of HCC regulated by DUBs.

USP2, USP11, USP22, USP30 and STAMBPL1 play a role in tumor activity by regulating lipid metabolism of HCC cells. These enzymes are crucial in the development of HCC associated with NASH, as they are involved in the regulation of disease progression and, due to their role in tumorigenesis, serve as potential diagnostic biomarkers and therapeutic targets.

Tumor cell glycolytic reprogramming is an important field in cancer research, a process often associated with the Warburg effect, whereby tumor cells tend to produce energy through glycolysis rather than oxidative phosphorylation, even in the presence of ample oxygen^212^. In the tumor microenvironment, glycolytic reprogramming is related to the proliferation and invasiveness of tumor cells. USP5, USP11, USP14, USP22, USP29 and USP48 promote tumor development by regulating glycolysis in HCC, and targeting these three deubiquitinating enzymes to inhibit glycolysis in HCC presents a new approach to suppress tumor growth.

PDL1 plays a crucial role in the tumor microenvironment, where tumor cells express PDL1 to inhibit the activity of T cells, thereby achieving immune evasion^213^, ^214^. USP22 and MINDY1 maintain the stability of PDL1 through the process of deubiquitination. The expression levels of USP22 and MINDY1 may indicate the sensitivity to PDL1 inhibitors, and inhibitors of USP22 or MINDY1 may enhance the efficacy of PD-1/PDL1 inhibitors and reduce drug resistance.

The MAPK, Hh, Wnt, Hippo and mTOR signaling pathways play crucial roles in the occurrence, development, invasion, and metastasis of HCC. The abnormal activation of these pathways not only promotes the proliferation and survival of tumor cells but also affects tumor invasion and metastasis by regulating the tumor microenvironment. Some DUBs are involved in the regulation of the activation of these signaling pathways. USP4 and USP12 promote the activation of the MAPK signaling pathway. USP44 inhibits the progression of hepatocellular carcinoma by suppressing the Hh signaling pathway. β-catenin, as a core regulatory factor of the canonical Wnt signaling pathway, has its protein levels regulated by USP8, USP9X, USP25, and USP39. In contrast, USP3, USP14, and JOSD2 do not regulate the Wnt signaling pathway by modulating β-catenin. USP1 and USP7 are involved in the regulation of the Hippo pathway, while USP10, USP19, and USP40 regulate the protein levels of YAP, a downstream effector of the Hippo pathway. The protein stability of mTOR is regulated by USP10, USP11, and USP22. Therefore, targeting these deubiquitinating enzymes represents an important therapeutic strategy with significant clinical implications.

EMT is a dynamic process wherein epithelial cells lose their polarity and cell-cell adhesion while gaining migratory and invasive properties characteristic of mesenchymal cells. This transformation plays a pivotal role in tumor invasion and metastasis^215^. EMT regulation involves a complex interplay of multiple signaling pathways, which are increasingly recognized as therapeutic targets. In this review, we summarize the mechanistic roles of USP4, USP5, USP9X, USP11, USP14, USP39, and BAP1 in modulating EMT and discuss their potential as novel targets for suppressing HCC metastasis.

From the existing research results, it can be seen that the abnormal expression of DUBs is closely related to drug resistance. Sorafenib and lenvatinib are currently first-line treatment drugs for HCC, but their therapeutic effects are limited and prone to drug resistance. In HCC cells and tumors resistant to lenvatinib, USP14 is significantly upregulated, and mutations in OTUD3 are associated with sorafenib resistance. The abnormal expression of USP8, USP29, and USP39 is also associated with sorafenib resistance. Additionally, the abnormal expression of USP8 and USP9 may be one of the reasons for HCC cells' resistance to doxorubicin. Furthermore, knocking down USP27 makes HCC cells more sensitive to 5-FU. The development of DUB inhibitors and their combination with current antitumor drugs is one of the promising directions for future research.

Ferroptosis is a form of programmed cell death that is dependent on iron and reactive oxygen species (ROS), characterized by the accumulation of lipid peroxides within cells, which ultimately leads to the disruption of cell membrane integrity and cell death^216^. Among DUBs, USP5 and USP8 inhibit ferroptosis in HCC, thereby promoting the progression and drug resistance of HCC. Developing specific inhibitors of USP5 and USP8 represents a potential anti-tumor strategy, especially for HCC that is resistant to conventional treatments.

This review summarizes 12 DUBs associated with drug resistance in HCC. Among these, USP3, USP8, USP9X, USP20, and CYLD promote HCC resistance to conventional chemotherapeutic agents (e.g., doxorubicin, platinum-based drugs). Additionally, USP7, USP8, USP24, USP29, and USP39 confer resistance to sorafenib, while USP14 and USP28 contribute to lenvatinib resistance. To better illustrate the roles of these DUBs in HCC drug resistance mechanisms, we have provided a schematic summary in Table 2.

DUBs play a crucial role in the pathogenesis of tumors. The development of targeted drugs and small-molecule inhibitors is advancing rapidly, representing a key focus of current and future research. Although most inhibitors remain in preclinical research, some drugs have already entered clinical trials. Significant progress has been made in the development of USP1 inhibitors, with two compounds advancing to Phase I clinical studies: KSQ-4279 (NCT05240898) and ISM3091 (NCT05932862), both targeting advanced solid tumors. VLX1570, a small-molecule inhibitor targeting USP14 and UCHL5, was the only DUB inhibitor to reach Phase II clinical trials (NCT02372240) for the treatment of multiple myeloma. However, the trial was terminated due to drug toxicity issues, highlighting the challenges in developing deubiquitinase inhibitors and underscoring the need for further research. In Table 1, we systematically summarize the current progress in the development of small-molecule inhibitors directed against DUBs.

Current research suggests that OTUB1, ATXN3, and JOSD1 promote the development of HCC, but the regulatory mechanisms of these three in HCC have not yet been elucidated in the literature. The research on the role of DUBs in the pathogenesis and treatment of HCC still has significant room for development. The development of targeted drugs and the clinical application of small molecule inhibitors will also become hot topics in future research. These can provide new ideas and research directions for the treatment of HCC in the future.

## Figures and Tables

**Figure 2:**
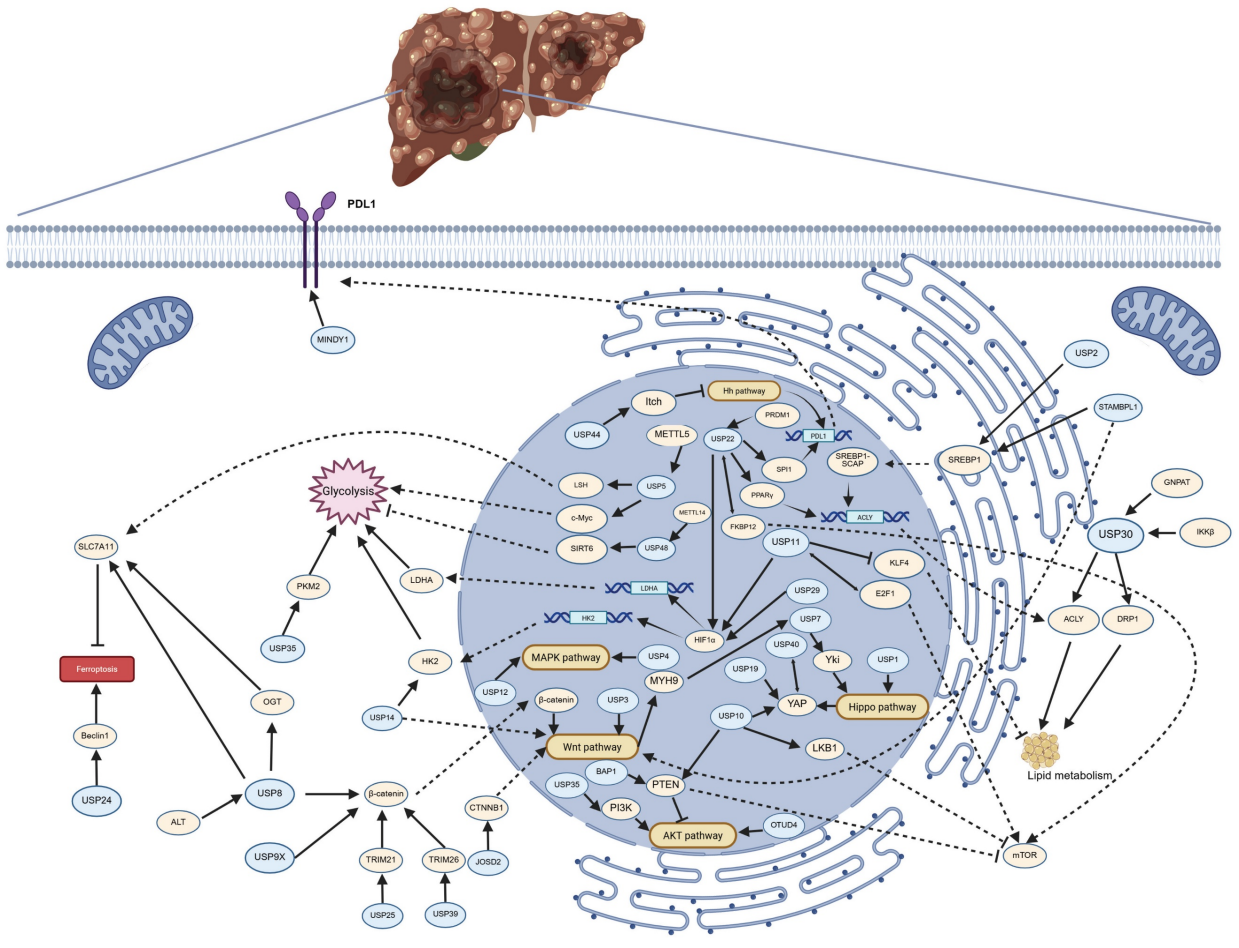
** The pathways regulated by deubiquitinating enzymes (DUBs) in the progression of hepatocellular carcinoma (HCC).** The figure illustrates the pathways by which DUBs regulate glycolysis, lipid metabolism, ferroptosis, PDL1 synthesis, and multiple signaling pathways in HCC. Arrows (→) indicate promotion or activation, while blocked lines (┫) denote inhibition or suppression. Created in https://BioRender.com.

**Table 1:** Roles of deubiquitinating enzymes (DUBs) in hepatocellular carcinoma (HCC) and their inhibitors.

DUBs family	DUBs	Mechanism pathway	Action	Article	Inhibitors
USP	USP1	USP1/Hippo pathway	Progression	32	KSQ-4279
		USP1/CDK5	Progression	34	ISM3091 FT-3171 SP-002
	USP2	USP2a/RAB1A	Progression	37	Q15
		USP2/SREPB1/2	Lipid metabolism	38	COH29 LCAHA 6TG
	USP3	USP3/β-catenin	Proliferation/Apoptosis	39	NC114 U4-I05
	USP4	USP4/CypA/MAPK pathway	Growth/Migration/Invasion	40	Vialinin A
		USP4/TGFR-1/TGF-β pathway	EMT/Metastasis	41	U4-I05
		USP4/Snail1	EMT	42	
	USP5	USP5/SLUG	Proliferation/Metastasis/Invasion/EMT	43	WP1130
		Hpn/USP5/p14(ARF)-p53 pathway	Apoptosis	45	PYR-41 Formononetin
		USP5/LSH/SLC7A11	Ferroptosis	44	Mebendazole
		CREB1/P300/METTL5/USP5/c-Myc	Glycolysis	46	USP5-IN-1
	USP7	USP7/Yki/Hippo pathway	Progression	50	Compound 19
		USP7/TMEM43/VDAC1	Progression	51	HBX28528
		USP7/YY1	Proliferation/Metastasis/EMT	52	HBX41108
		USP7/BTF3	Proliferation/Metastasis/Invasion	53	GNE-6676
		USP7/TRIP12/p14	Progression	48	I-56/57
		USP7/TRIM27/STAT3	Progression	54	Compound-23/25/28
		miR‑205/USP7	Proliferation	47	
		ENKUR/β-catenin/c-Jun/MYH9/USP7	Proliferation/EMT	55	FT827/671
		METTL3/USP7	Proliferation/Metastasis/Invasion	56	
		FEN1/USP7/MDM2/p53	Metastasis/Invasion/EMT	57	
	USP8	USP8/RTKs	Apoptosis	62	ML364 Compound 22c DC-U43-10 DC-U4106
		USP8/β-catenin	Tumorigenesiss	63	
		USP8/OGT/SLC7A11	Ferroptosis	65	
		USP8/TRAF6/ NF-ΚB	Autophagy	66	
	USP9X	USP9X/β-catenin	Proliferation	67	WP1130
		MiR26b/USP9X/Smad4/TGF-β	EMT	68	EOAI3402143
		MiR-26b/USP9X/p53	Autophagy	70	FT709
		LNC473/USP9X/survivin	Proliferation/Invasion	71	
	USP10	USP10/PTEN/mTORC1	Progression	72	Spautin-1
		USP10/LKB1/mTOR	Progression	73	Quercetin
		USP10/Smad4	Metastasis	74	DZNep
		USP10/YAP/p53	Proliferation	75	HBX19818
		USP10/PLAGL2	Progression	79	Wu-5
	USP11	USP11/NF90	Proliferation/Metastasis	81	Mitoxantrone
		USP11/E2F1/ERK/mTOR	Metastasis/Autophagy	82	
		USP11/Eef1A1/SP1/HGF	EMT/Metastasis	83	
		USP11/KLF4	Lipid metabolism	84	
		USP11/HIF-1α/LDHA	Glycolysis/Proliferation/Metastasis	85	
	USP12	USP12/The cyclin dependent kinase 1/cyclinB1	Proliferation	86	—
		USP12/p38/MAPK pathway	Autophagy	86	
		USP12/c-Myc	Progression	87	
	USP13	USP13/c-Myc	Progression	88	Spadin-1
		USP13/TLR4/MyD88/NF-κB	Proliferation/EMT/Migration/Invasion	89	BK50118C
	USP14	USP14/Wnt/Notch1 signaling pathway	Endoplasmic reticulum/ Progression	91	VLX1570 IU1
		USP14/ HIF1α	Proliferation/Invasion/Migration/Vascular Mimicry formation	92	b-AP15 PtPT
		USP14/HK2/AKT/P62	Proliferation/Invasion/Autophagy/Glycolysis	93	
	USP15	-	Gene expression/Cell cycle/DNA repair	95	USP15-IN-1
			Proliferation/Autophagy	96	
	USP16	Ct-HBX/USP16	Progression	102	—
	USP18	USP18/BCL2L1	Proliferation	103	—
	USP19	USP19/YAP	Proliferation/Migration	104	Example 142
	USP20	ATR/USP20/SLC7A11	Ferroptosis	105	GSK2643943A
	USP21	USP21/MEK2	Progression	106	BAY-805 ZINC02422616
		hsa_circ_0039053/USP21	Progression	107	
	USP22	USP22/HIF1α	Stemness/Glycolysis	108	Baohuoside I
		USP22/ FKBP12/ mTORC1	Tumorigenesis/Progression/Autophagy	109	Morusin
		USP22/PPARγ/ACLY and ACC	Tumorigenesis/Lipid metabolism	110	Chrysin
		PRDM1 /USP22/SPI1/PDL1	Tumor immunity	111	USP22i-S02 Pirarubicin
	USP24	USP24/TRAF2	Progression	115	WP1130 EOAI3402143 NCI677397
		MiR-21-5p/USP24/SIRT7	Progression	116	
		USP24/Beclin1	Proliferation/Migration/Ferroptosis/ Autophagy	117	
	USP25	USP25/TRIM21/Wnt/β-catenin signaling pathway	Proliferation/Migration/Invasion/EMT	119	PR619 AZ1 Vismodegib Compound 9p FT206
	USP26	HBx/USP26/SIRT1	Proliferation/Apoptosis	120	—
	USP27	USP27/Cyclin E	Growth/Migration/Invasion	125	—
		USP27/SETD3	Proliferation/Progression	126	
	USP28	miR-363-3p/USP28/c-Myc	Progression	129	CT113 AZ1
		miR-216b/USP28	Progression	128	
	USP29	USP29/HIF1α/HK2	Glycolysis	133	—
	USP30	GNPAT/USP30/DRP1	Lipid metabolism/Tumorigenesis	134	MTX652
		IKKβ/USP30/ACLY	Lipid metabolism/Tumorigenesis	135	MF094 ST-539 USP30_inh_
	USP33	USP33/SP1/c-Met	Invasion/Metastasis	140	—
	USP35	USP35/PKM2	Proliferation/Migration/Invasion	141	—
		USP35/ABHD17C	Proliferation/Apoptosis/Migration/Invasion	142	
		USP35/PI3K/AKT signaling pathway	Proliferation/Apoptosis/Migration/Invasion	142	
	USP39	USP39/SP1	Proliferation	145	—
		USP39/TRIM26/ZEB1	EMT/ Proliferation/Metastasis	146	
		USP39/TRIM26/β-catenin	Proliferation/Metastasis	147	
		USP39/FoxM1/ PLK1 and cyclin B1	Proliferation	143	
		SIRT7/USP39	Proliferation/Tumorigenesis	149	
		DNAAF5/USP39/PFKL	Proliferation	150	
	USP40	USP40/Claudin1	Proliferation/Migration/Stemness	151	—
		USP40/YAP	Progression	152	
	USP44	USP44/Itch/Gli1/PDL1	Progression	154	—
	USP46	USP46/MST1	Proliferation/Metastasis	155	—
		MiR-27a-3p/USP46	Proliferation	156	
	USP48	USP48/Gasdermin E	Pyroptosis	157	—
		Mettl14/USP48/SIRT6	Glycolysis/Malignancy	158	
	USP49	HSF1/HLNC1/USP49	Progression	159	—
	USP53	USP53/CYCS	Apoptosis	162	—
	CYLD	CYLD/IκB-α	Apoptosis	163	Dihydromyricetin
		CYLD/JNK/c-MYC	Proliferation	164	
		miR-362/CYLD	Growth/Metastasis	165	
		miR-501/CYLD	Proliferation	167	
		miR-922/CYLD	Proliferation	168	
		SOX2/EGFR/miR-222-5p/CYLD	Proliferation/Migration/Invasion	169	
OTU	OTUB1	-	Progression	170	Compound 61
	OTUD3	OTUD3/ACTN4	Proliferation/Metastasis	171	—
		OTUD3/ eIF2α/ISR	Progression	172	
	OTUD4	OTUD4/AKT signaling pathway	Proliferation/Metastasis/Invasion/Autophagy	176	—
	OTUD5	OTUD5/ SLC38A1	Proliferation	177	—
	OTUD7B	OTUD7B/p53/PUMA/BAX	Progression/Autophagy	178	—
	TRABID	TRABID/DDB2	Proliferation	179	—
UCH	UCH37	UCH37/GRP78	Progression	180	b-AP15
		UCH37/PRP19	Metastasis/Invasion	181	
	BAP1	BAP1/PTEN/AKT/GSK-3β/Snail pathway	Proliferation/Invasion/EMT	182	iBap
JOSD2	JOSD2	JOSD2/CTNNB1/Wnt pathway	Progression	197	HY041004
MINDY	MINDY1	MINDY1/PDL1	Tumor immunity	200	—
JAMM	STAMBPL1	STAMBPL1/Wnt/β-catenin	Proliferation	208	CSN5i-3
		STAMBPL1/SREBP1	Lipid metabolism	208	

**Table 2:** DUBs Involved in HCC Drug Resistance.

DUBs	Resistant Drug(s)	Experimental Validation	Article
USP3	Conventional chemotherapeutic drugs	*In vitro*	39
USP7	Sorafenib	*In vitro/* Patient-derived xenograft	217
USP8	Doxorubicin/ Sorafenib	*In vitro/* Cell Line-Derived Xenograft	62
USP9X	Doxorubicin	*In vitro/* Cell Line-Derived Xenograft	70, 218
USP14	Lenvatinib	*In vitro/* Cell Line-Derived Xenograft	94
USP20	Oxaliplatin	*In vitro/* Cell Line-Derived Xenograft	105
USP24	Sorafenib	*In vitro/* Cell Line-Derived Xenograft	117
USP28	Lenvatinib	Cell Line-Derived Xenograft	130
USP29	Sorafenib	*In vitro/* Patient-derived xenograft	133
USP39	Sorafenib	*In vitro*	150
CYLD	Doxorubicin/ 5-Fluorouracil/ Cisplatin	*In vitro*	163
OTUD3	Sorafenib	*In vitro/* Cell Line-Derived Xenograft	172

## Abbreviations

FEN1: Flap endonuclease 1
FoxM1: Forkhead box M1
HBx: Hepatitis B virus X protein
HCC: Hepatocellular carcinoma
HK2: Hexokinase 2
ICC: Intrahepatic cholangiocarcinoma
ISR: Integrated stress response
JAMM: JAMM/MPN domain-associated Zn-depend metalloproteases
MINDY: The motif interacting with ubiquitin (MIU)-containing novel DUB family
MJD: Machado-Joseph domain-containing protease
mTORC1: Mammalian target of rapamycin complex 1
NASH: Non-alcoholic steatohepatitis
OGT: O-GlcNAc transferase
OUT: Ovarian tumor protease
PAK1: P21-activated kinase 1
PDL1: Programmed Cell Death Ligand 1
PFKL: Phosphofructokinase L
RTK: Receptor tyrosine kinase
SIRT7: Sirtuin 7
TMEM43: Transmembrane protein 43
TRABID: TRAF-binding domain-containing protein
TRAF2: Tumor Necrosis Factor Receptor-Associated Factor 2
TRIP12: Thyroid hormone receptor-interacting protein 12
UCH: Ubiquitin C-terminal hydrolase
USP: Ubiquitin-specific protease
YAP: Yes-associated protein
YY1: Yin-yang-1
ZEB1: Zinc-finger E-box-binding homeobox 1
